# EmotionCast: An Emotion-Driven Intelligent Broadcasting System for Dynamic Camera Switching

**DOI:** 10.3390/s24165401

**Published:** 2024-08-21

**Authors:** Xinyi Zhang, Xinran Ba, Feng Hu, Jin Yuan

**Affiliations:** School of Information and Communication Engineering, Communication University of China, Beijing 100024, China; zxinyi@cuc.edu.cn (X.Z.); fenghu@cuc.edu.cn (F.H.); yuanjin@cuc.edu.cn (J.Y.)

**Keywords:** emotion recognition, intelligent broadcasting, multimodal data processing

## Abstract

Traditional broadcasting methods often result in fatigue and decision-making errors when dealing with complex and diverse live content. Current research on intelligent broadcasting primarily relies on preset rules and model-based decisions, which have limited capabilities for understanding emotional dynamics. To address these issues, this study proposed and developed an emotion-driven intelligent broadcasting system, EmotionCast, to enhance the efficiency of camera switching during live broadcasts through decisions based on multimodal emotion recognition technology. Initially, the system employs sensing technologies to collect real-time video and audio data from multiple cameras, utilizing deep learning algorithms to analyze facial expressions and vocal tone cues for emotion detection. Subsequently, the visual, audio, and textual analyses were integrated to generate an emotional score for each camera. Finally, the score for each camera shot at the current time point was calculated by combining the current emotion score with the optimal scores from the preceding time window. This approach ensured optimal camera switching, thereby enabling swift responses to emotional changes. EmotionCast can be applied in various sensing environments such as sports events, concerts, and large-scale performances. The experimental results demonstrate that EmotionCast excels in switching accuracy, emotional resonance, and audience satisfaction, significantly enhancing emotional engagement compared to traditional broadcasting methods.

## 1. Introduction

Live video directing, which refers to the process of directing live events such as concerts, broadcasts, or productions in real-time, involves the control of various aspects of production, such as camera angles, volume, and lighting [1]. Real-time directing is a complex and precise task that typically relies on a professional director’s expertise. These professionals must switch between multiple camera feeds quickly and adjust them based on their living situation. This requires high levels of skill, rapid response, and precise judgment. With the complexity and richness of live content, the limitations of traditional live-directing methods have become apparent. Directors must maintain intense concentration for extended periods, which can result in fatigue and decision-making errors, particularly during long broadcasts. This issue is particularly pronounced during prolonged live events. Furthermore, directors must make accurate judgments and the camera switches in a brief time, which places tremendous demands on their reaction speed and operational accuracy.

In recent years, the broadcasting industry has progressively embraced intelligent production methods, with artificial intelligence (AI) technology achieving numerous successful applications in news media [2]. The integration of AI technology into TV production has demonstrated significant potential in terms of enhancing production efficiency, minimizing human error, and reducing costs. Automated intelligent directing systems have attracted substantial interest from researchers and industry professionals worldwide. These systems can significantly alleviate directors’ workloads and enhance their production efficiency. In contrast to human operators, intelligent systems can function continuously and efficiently and are unaffected by fatigue, thereby reducing decision-making errors. Moreover, these systems decrease the reliance on professional directors, thereby lowering training and labor costs while optimizing equipment utilization.

With the development of more human-like intelligent agents, artificial intelligence must consider emotion, the most basic spiritual need in human interaction [3]. As a crucial task in the field of natural language processing (NLP), sentiment analysis aims to identify the affective tendencies of each aspect entity in textual utterances or other modal data [4]. Different modalities reflect sentiment with various intensities, and they may possess consistent or independent information [5]. If the connection between various modalities can be mined, the accuracy of sentiment analysis will be further improved [6]. Therefore, multimodal sentiment analysis (MSA) is important for quickly and accurately understanding people’s attitudes and opinions about an event. MSA aims to improve the sentiment analysis of text modality by jointly determining the sentiment of the subject through multiple modalities including textual, visual, and auditory [7].

However, researchers have found that the performance in previous MSA tasks has been overly dependent on text modality [5], limiting the understanding of emotional changes. Prior studies in this area often rely on feature concatenation to obtain joint representations, failing to fully exploit interactive patterns to ensure consistency and differentiation across different modalities [8]. In addition, intelligent directing systems are plagued by challenges in handling complex scenarios, owing to insufficient contextual understanding, singular decision-making processes, and a lack of human touch. Current research predominantly relies on pre-set rules and model-based decisions, focusing more on technical precision while neglecting the audience’s need for emotional and atmospheric engagement. This results in a lack of emotional resonance in live broadcast content. To address these issues, this study proposes an emotion-driven intelligent directing system called EmotionCast.

EmotionCast aims to simulate the switching strategies of human directors by integrating advanced deep-learning algorithms and emotion recognition technologies, thereby providing efficient and intelligent live broadcast operations for semi and fully automated production. The system employs sensor devices to capture changes in the live environment and audience reactions, processing multimodal input information such as facial expressions, body postures, and vocal tone in real-time. Based on the current emotional content, it dynamically adjusts camera switching strategies to ensure that the cameras consistently capture the most expressive shots, thus enhancing the connection between the audience and the live broadcast content.

The remainder of this paper is organized as follows. Section 2 reviews related works, including existing intelligent directing technologies and emotion recognition research, thus highlighting the potential of emotion recognition to enhance live content quality and audience engagement. Section 3 provides a detailed description of the design and implementation of the EmotionCast system, including emotion recognition, multimodal data processing, and dynamic planning modules. Section 4 outlines the experimental setup of the EmotionCast system including the experimental environment, evaluation metrics, testing procedures, data collection, and analysis methods. In Section 5, the performance evaluation of the system based on the test results is presented and the impact of emotion-driven camera switching along with the limitations encountered during implementation are discussed. Finally, we conclude with the application prospects of the EmotionCast system in modern media production and propose future research directions.

The primary contributions of this study are as follows:

- EmotionCast, an emotion-driven intelligent directing system, assists directors in overcoming real-time camera switching challenges posed by multimodal data scenarios.
- To address the deficiencies of traditional directing systems in emotion recognition and response, EmotionCast extracts emotional features from various signal sources, automatically adjusts camera angles to respond to the live context and ensures emotional consistency in camera-switching decisions.

## 2. Related Work

Intelligent directing aims to leverage AI to combine emotion analysis with video-switching, thereby enhancing the quality of live content and increasing audience engagement. In this subsection, we first provide an overview of the current state of intelligent directing systems and the application of AI to existing directing systems. Next, we review how automated switching technology assists directors in the real-time selection of the most appropriate camera angles. Finally, we discuss the previous research on the use of multimodal data for emotion analysis.

### 2.1. Intelligent Directing Systems

By integrating AI technology, broadcasters can offer audiences a more dynamic and immersive experience, thereby potentially increasing viewer engagement and expanding the reach of live events [9]. Intelligent systems signify the future of automated media production and showcase the substantial potential of AI and machine learning in enhancing the efficiency and adaptability of live broadcasts. Global newsrooms have begun to increasingly adopt AI for news automation [2], and intelligent directing systems are becoming increasingly prevalent. These systems leverage deep learning and computer vision techniques to automatically identify and analyze individuals and events in video streams. This facilitates autonomous adjustments of camera angles and switching strategies.

Previous studies proposed several noteworthy switching strategies. For example, the ChunkyEdit system prioritizes text-based editing to manage block-based video interview editing [10]. This approach is particularly suitable for live events requiring rapid responses and updates. Event-driven intelligent directing systems can automatically detect significant events during a live broadcast and dynamically adjust cameras and perspectives accordingly [11]. The study [12] utilizes the YOLOv8 algorithm to accurately identify and segment camera operators in live football video broadcasts. The IDirector system leverages machine learning to analyze visual and audio inputs in real time, thereby automating the camera switching and scene selection to mimic the decision-making process of human directors. This reduces the need for manual intervention and enhances the viewer experience [13,14].

Although these studies provide a technical foundation for the development of intelligent directing systems, they often overlook the practical requirements of directors and media professionals in the operation and management of these systems.

### 2.2. Automated Camera Switching

The application of multi-camera systems has demonstrated significant progress and broad potential. By utilizing gaze data, multi-camera systems can achieve automated camera selection and editing, thus allowing operators to choose and edit virtual camera perspectives in real time. This significantly enhances flexibility and interactivity in video production [15,16]. For instance, a study [17] analyzes the focal areas of cameras to identify important content within a scene, thereby facilitating a method for automatic editing of footage from multiple social cameras. Another study [18] utilized intelligent algorithms to facilitate the coordination of multi-camera systems. This method facilitated automatic selection of the best camera angles to ensure high-quality footage and comprehensive event coverage.

Multi-camera systems have also been applied to various practical scenarios. In virtual lecture environments, systems utilize attention mechanisms to identify interaction points between teachers and students. They automatically select the most appropriate shots from multiple cameras to optimize the teaching and learning experiences [19]. In real-time video production, systems such as Cinemassist significantly simplify the direction of workflow by automating composition and camera adjustment [20]. For dialogue-driven scenes, computational video editing techniques enhance the coherence and visual appeal of videos through automation [13]. Live broadcasts of sports events leverage decision tree algorithms to analyze key events and player positions. They automatically select the best camera angles to improve the viewing experience and reporting accuracy [21]. Further, Ranjan et al. demonstrated that effective editing rules could be established using cues such as speaker detection, gestures, and head direction, thereby improving the editing of group meetings [22].

Although current systems can perform camera switching based on preset rules and algorithms, they often lack a deep understanding of complex human emotions and non-verbal communication. Consequently, this may fail to fully capture the director’s intentions and audience expectations.

### 2.3. Emotion Recognition Technologies

Emotion recognition enables machines to understand human emotions and has significant potential for various applications [23]. For instance, the highly efficient convolutional neural network (CNN) model designed in a previous study [24] can quickly and accurately identify the emotional states of individuals from video streams. Moreover, technological advancements have significantly improved the efficiency and accuracy of emotion recognition. Features extracted from facial images can be used to identify emotional states [25]. Through an analysis of key landmarks in facial images captured by cameras, researchers can determine user emotional states [26]. Further, they can perform real-time emotion detection through quantitative facial motion analysis [27]. Real-time facial emotion recognition technology, particularly in visualization systems, has demonstrated effective emotional state monitoring capabilities [28].

Regarding auditory analysis, a study [29] discussed the effectiveness and applications of various deep learning models in parsing and recognizing emotional expressions in speech. Continuous emotion prediction from uniformly segmented speech recordings highlights the value of audio data in emotion recognition [30]. DNN-based models can learn and recognize emotions directly from raw speech signals without traditional acoustic feature extraction steps [31].

In addition, multiple perceptual modalities have been used for emotion recognition. Based on deep learning architectures, a study [32] focused on hierarchical video summarization and facial tracking, whereas another study [23] emphasized the combination of voice and facial expressions. Hossain et al. [33] employed emotional big data from audio and video, and Hasan et al. [34] automatically generated video highlights. The MSEVA system in a study [35] integrated visual, audio, and textual data, similar to how other studies [36,37] combined facial expressions, vocal tones, body gestures, and physiological signals into multimodal emotion recognition systems. A study [6] utilized multi-modal cross-attention to fuse the extracted features from the text and image, and we classify the output to determine the emotional polarity. Another study [38] provides a comprehensive review of emotion recognition and detection methods, particularly discussing the technologies and challenges of multimodal emotion recognition. These advances enhance the accuracy and application scope of emotion recognition and offer new directions and insights for the future development of this technology. Despite the impressive performance of emotion-driven systems in various fields, their application to intelligent directing remains underexplored.

The EmotionCast system addresses these three issues. Through the combination of visual, auditory, and textual data for multimodal emotion recognition, the system transitioned from simple script-driven operations to more complex context-aware systems. This facilitated the intelligent directing system in responding to simple commands and understanding and reacting to complex human emotions and scene changes. Consequently, this enhanced the emotional impact and appeal of the content, evoked emotional resonance, and provided a more efficient and humanized directing solution.

## 3. EmotionCast

**Figure 1:**
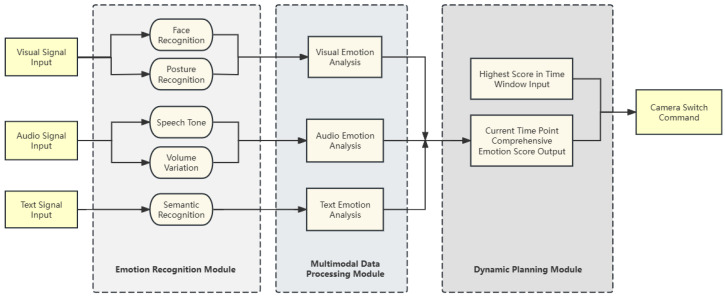
Architecture of the EmotionCast system illustrating the three main modules: emotion recognition, multimodal data processing, and dynamic planning.

This section describes the design and implementation of EmotionCast. The system comprised three main modules: an emotion recognition module, a multimodal data processing module, and a dynamic planning module, as shown in Figure 1. The emotion recognition module collected real-time video and audio data from multiple cameras and employed deep learning algorithms to analyze facial expressions, vocal tones, and other cues to detect emotions. Next, the multimodal data processing module combined visual, auditory, and textual analyses to generate emotional scores for each camera. Finally, the dynamic planning module employed state-transition equations to determine optimal camera switching.

### 3.1. Emotion Recognition Module

The emotion recognition module extracted emotional features from various signal sources and generated emotional scores. This module primarily processed visual, auditory, and textual signals.

#### 3.1.1. Visual Signal Processing

In the EmotionCast system, we employed facial expression analysis and posture recognition technologies. First, we used the deep learning model OpenFace to detect and recognize faces, capture subtle facial changes such as smiles and frowns, and analyze individual emotional states. In addition to facial expressions, we utilized OpenPose posture-recognition technology to detect key human points and interpret body language, such as gestures and overall body movements, to infer emotional states. For instance, an excited person may perform more hand movements, whereas a dejected person may display negative postures, such as looking down. This submodule combines the results of facial and posture recognition to calculate the visual emotional score, reflecting the emotional intensity expressed by the characters’ facial expressions and movements captured by the camera. The visual-emotional score at time *t* for camera *c* is denoted as $E_{V}\left(t,c\right)$, where *t* represents the current time point and *c* represents the current camera.

#### 3.1.2. Audio Signal Processing

The audio signal processing submodule began by extracting audio features using OpenSMILE and analyzing the speaker’s tone and pitch to identify emotional states. Subsequently, it examined variations in volume to detect emotional states, such as fluctuations in volume and sudden increases or decreases, often reflecting intensity or changes in emotions. Finally, the analyses of vocal tone and volume variations were combined to calculate the audio-emotional score, which represented the emotional intensity conveyed through the sound in the camera footage. The audio-emotional score at time *t* for camera *c* is denoted as $E_{A}\left(t,c\right)$, where *t* represents the current time point and *c* represents the current camera.

#### 3.1.3. Text Signal Processing

The text signal processing submodule identified emotional states by analyzing real-time captions and other textual inputs. In EmotionCast, we employed bidirectional encoder representations from Transformers (BERT) as tools for text emotion recognition. Initially, the input text signals were preprocessed, including tokenization and stop-word removal. The preprocessed text was then subjected to sentiment analysis using the pre-trained BERT model to extract emotional features. Based on the emotional feature output of the BERT model, the text-emotional score was calculated to reflect the emotional intensity conveyed by the text content. The text-emotional score at time *t* for camera *c* is denoted as $E_{T}\left(t,c\right)$, where *t* represents the current time point and *c* represents the current camera.

### 3.2. Multimodal Data Processing Module

In the multimodal data processing module, we integrated emotional signals from various sources to generate a comprehensive emotional score. Using a weighted average method, we combined the visual, auditory, and textual emotional scores to calculate the comprehensive emotional score E(t,c) for camera *c* at time point *t*, which is implemented in Equation (1):

(1) $$E\left(t,c\right)=w_{V}E_{V}\left(t,c\right)+w_{A}E_{A}\left(t,c\right)+w_{T}E_{T}\left(t,c\right)$$

The weights $w_{V},w_{A}$, and $w_{T}$ represent the relative importance of the visual, auditory, and textual emotional scores, respectively, in the emotion analysis. These weights can be adjusted based on the actual requirements.

To ensure that the emotional scores were within a reasonable range and allow the system to dynamically adjust its sensitivity to different modalities, we included a normalization step. This ensured that the scores from each modality were on the same scale before their combination, which is implemented in Equation (2):

(2) $$E\left(t,c\right)=\frac{w_{V}E_{V}\left(t,c\right)}{\max_{c^{\prime }}E_{V}\left(t,c^{\prime }\right)}+\frac{w_{A}E_{A}\left(t,c\right)}{\max_{c^{\prime }}E_{A}\left(t,c^{\prime }\right)}+\frac{w_{T}E_{T}\left(t,c\right)}{\max_{c^{\prime }}E_{T}\left(t,c^{\prime }\right)}$$

The term $\max_{c^{\prime }}E_{X}\left(t,c^{\prime }\right)$ represents the maximum score for modality *X* across all cameras at time *t*. This normalization ensured that the scores from the different modalities were comparable when weighted. Finally, the comprehensive emotional score E(t,c) was passed to the state transition module.

### 3.3. State Transition Module

In the state-transition module, we combined the current emotional score at time *t* with the optimal scores from the preceding time window to calculate the current frame score and thus select the optimal camera angle. First, we defined a window of size *W*, which covered the range of *W* time units preceding the current time *t*. We then selected the highest score within this window as an influencing factor and used the state transition equation to compute the optimal score at the current time, which is implemented in Equation (3):

(3) $$S\left(t,c\right)=w_{t}E\left(t,c\right)+w_{k}\max_{t-W\leq k\leq t}S\left(k,c\right)$$

where $w_{t}$ and $w_{k}$ are weighting coefficients, with $w_{k}$ specifically used to balance the influence of the scores from previous time points while maintaining the continuity of the optimal strategy. Further, S(t,c) is the total score at time *t* for camera *c*, and $\max_{t-W\leq k\leq t}S\left(k,c\right)$ is the highest score for camera *c* within the *W* time units preceding time *t*.

In addition, the choice of the sliding window *W* must consider the dynamic nature of the live content. The selection of a smaller window renders the camera switching more responsive. If live content changes rapidly and exhibits significant emotional fluctuations, such as in sports events or live concerts, a smaller window size may be necessary to promptly capture emotional changes and quickly respond to important events. Conversely, a larger window results in a smoother camera transition. For more stable or narrative content, such as interviews or documentaries, wherein emotional changes are slower, a larger window can be chosen to enhance emotional continuity.

Through this process, EmotionCast can integrate visual, auditory, and textual information to perform emotion analysis based on multimodal data. The system selects the camera feed that best represents the current emotional state at each time point, by combining the emotional score of the current camera with the optimal state from the previous time window. This optimizes the emotional expression and audio–visual quality of live broadcasts, thereby enhancing viewer interaction and satisfaction.

## 4. Experimental Setup

**Figure 2:**
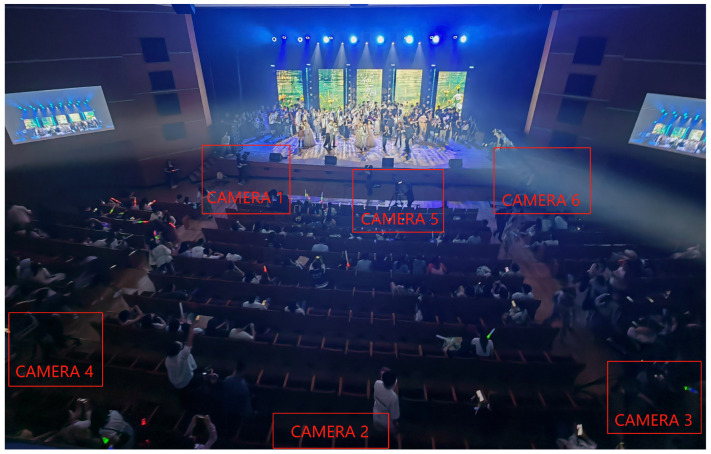
Distribution of the six camera setups in the live broadcast environment, showing their strategic positions to capture different angles of the event.

**Figure 3:**
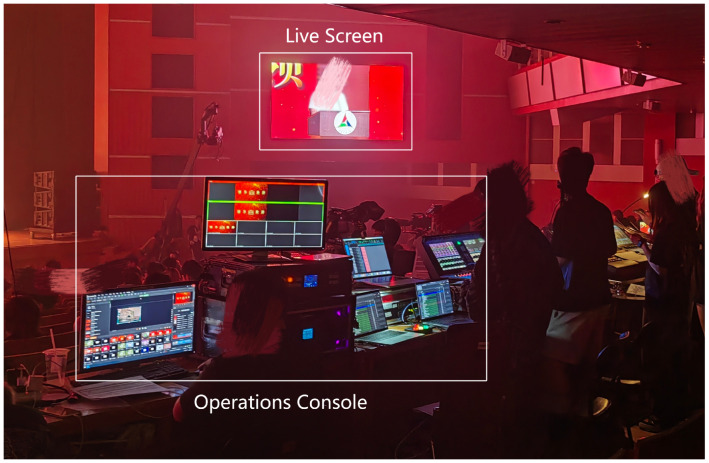
Setup of the backstage monitoring and operation equipment, illustrating the coordination and monitoring of live feeds from the six cameras by the directing team.

**Figure 4:**
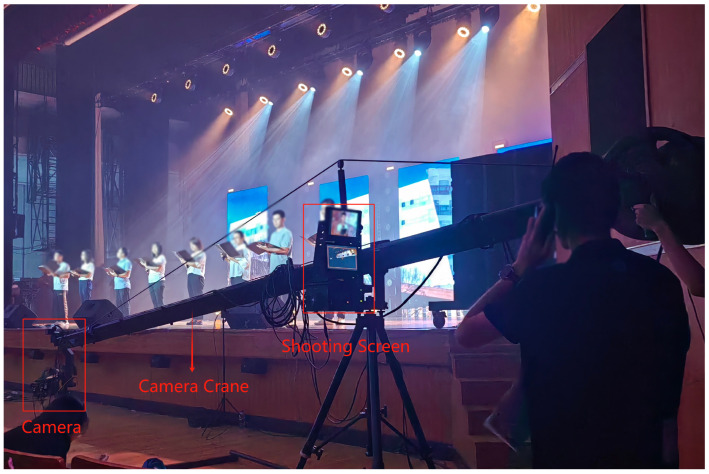
The camera equipment used for the live broadcast, highlighting the high-definition cameras and other related equipment deployed for capturing the event.

To comprehensively evaluate the performance of EmotionCast, we designed a subjective evaluation test. The test venue was a 1500-seat lecture hall at the Communication University of China during a live broadcast of the School of Information and Communication Engineering’s graduation ceremony. Six high-definition cameras were used to capture footage from different angles to ensure diverse and high-quality video data. High-fidelity microphones and mixers were used to capture and process live audio to ensure clear and accurate audio data. The entire event was broadcast using six camera channels, as shown in Figure 2. The audio and video materials for the EmotionCast system were sourced from the recordings of these six cameras. Figure 3 and Figure 4 show the broadcast center and filming equipment on-site, respectively.

During the live performances, we recorded the camera-switching moments directed by the on-site director, noting the exact timing and number of switches for each segment. Simultaneously, we asked participants to record the moments during the performance when they felt significant emotional fluctuations. We selected three segments from the entire event as test samples: a lyrical song performance, a fast-paced dance performance, and a dramatic skit. Following the live performance, we used EmotionCast to analyze the original video recordings, score different camera angles, and select the optimal switching points.

During the experimental phase, we labeled the videos directed by the human operator and EmotionCast as Video 1 and Video 2, respectively. The participants, unaware of the video order, watched both videos and completed a questionnaire. The questionnaire assessed their emotional fluctuations and subjective feedback, focusing on four evaluation metrics: smoothness, diversity, emotional resonance, and switching matching. Before the experiment began, the participants were thoroughly briefed on the meaning of each evaluation metric and the scoring criteria, with different dimensions used for each metric.

To gather comprehensive data, we conducted three rounds of testing: the first immediately after the live performance, the second on the following day, and the third two days later. The study involved 20 participants, aged between 20 and 30, including 10 males and 10 females. While we acknowledge that age may influence perception, we believe the diversity of our sample is sufficient to represent the views of a young adult audience for this research.

To evaluate the quality of the video generated by the switching system, we propose seven evaluation indexes. For metrics such as switching accuracy, switching frequency, and system stability, we collect and analyze objective data. Meanwhile, for smoothness, scene diversity, switching matching, and emotional resonance, we use questionnaires to gather subjective ratings from participants and analyze the results based on their feedback. Our research goes beyond traditional broadcasting methods by integrating emotional resonance as a key evaluative criterion. This approach ensures that the content does not merely switch based on arbitrary metrics but is finely tuned to enhance the viewer’s engagement by aligning with their emotional responses.

**Switching accuracy** refers to whether the transitions between shots in a video or live broadcast accurately capture key scenes, focus on important characters or events, and occur at appropriate moments to ensure continuity and audience comprehension. To measure switching accuracy, we reviewed the recorded footage and assessed whether the camera transitions aligned with the detected emotional states and the unfolding narrative. For each program, we documented the switching points for both EmotionCast and the human director, comparing the consistency of their results. This comparison allowed us to determine how many times EmotionCast and the human director successfully executed correct transitions in different segments of each program, thereby quantifying the accuracy of the switching.

**Switching frequency** refers to the number of camera switches within a given time period, reflecting the system’s responsiveness and the frequency at which it adjusts views based on emotional cues or scene changes. In this study, a camera switch is defined as any change in the main video frame, including cuts, fades, and dissolves between different camera angles or shots. To ensure data accuracy and consistency, researchers conducted three independent viewings and analyses of each program segment. During each viewing, researchers marked every camera switch and used a timer to record its precise timing. This process was applied to both the switches generated by EmotionCast and those made by human directors, allowing for a direct comparison.

For each program segment, we tallied the total number of switches, calculated the average time interval between switches based on the three independent observations, and computed the standard deviation of these intervals. To further ensure the reliability of the data, if the time interval difference between any two viewings exceeded 5%, a fourth viewing was conducted to resolve any discrepancies.

**System stability** refers to the consistent and continuous execution of switching operations, maintaining expected performance over extended periods. A stable system should preserve relative consistency in switching intervals across various program types and contexts, avoiding both excessive and insufficient switching while adapting to dynamic content changes. To evaluate stability, we use two primary metrics: the standard deviation of switching frequency and the range of switching intervals. The standard deviation reflects the variability in switching times, with lower values indicating more stable frequency. The interval range, defined as the difference between the longest and shortest intervals, represents overall switching consistency. By analyzing these metrics in conjunction with considerations of genre-specific characteristics and comparisons against manual operations, we can conduct a comprehensive and objective assessment of the system’s stability performance.

**Smoothness** refers to the overall fluidity experienced by viewers during a live broadcast, encompassing the smoothness of camera transitions and the continuity of playback. This metric primarily evaluates the natural flow and coherence of visual information, determining whether camera switches effectively guide the viewer’s gaze without causing jarring or uncoordinated visual disruptions. The evaluation is conducted through subjective ratings, where participants assess their viewing experience on a scale of 1 to 10, with 10 indicating the most seamless, natural, and uninterrupted switching experience. To reduce potential biases from subjective perceptions, we average the scores from multiple rounds of testing for the same program to determine the final smoothness score.

**Scene diversity** refers to the variety and variation within video or live content, including camera angle changes, scene transitions, and the use of visual effects. A diverse presentation of scenes enhances the comprehensive and accurate conveyance of information. Camera angle variation assesses how frequently and effectively different perspectives are utilized. Scene content changes emphasize the degree of variation between consecutive scenes. Visual effect diversity highlights the range of filming techniques employed, such as close-ups, wide shots, and tracking shots. Participants independently rate each of the three dimensions on a scale of 1 to 10, with 10 representing the highest level of richness and excellence in that dimension. We then calculate the average score for each dimension and use the overall average of these three dimensions as the final scene diversity score.

**Switching matching** refers to the degree of alignment between camera switches and the content of the program. It assesses whether the timing and angles of the camera switches appropriately correspond to scene changes, character movements, as well as the narrative pace and emotional tone. We evaluate switching compatibility based on two key dimensions: the alignment with scene or character changes and the alignment with the overall rhythm and atmosphere.

First, we assess the synchronization of camera switches with scene changes or dynamic character movements. For example, when a new character begins speaking or the scene changes, the camera should switch to an appropriate angle to highlight this change. Second, we evaluate the alignment of camera switches with the narrative progression, pacing, and tone of the program. For instance, in tense scenes, the frequency of camera switches typically increases, while in slow-paced, emotionally rich scenes, the switching becomes softer and slower. To ensure the accuracy of the evaluation, we independently measure each dimension, with participants rating from 1 to 10. The final switching compatibility score is then obtained by averaging the scores of the two dimensions.

**Emotional resonance** refers to the depth and intensity of emotional responses experienced by viewers while watching a program. It serves as a measure of the emotional connection established between the audience and the content. A high emotional resonance score indicates that the program effectively communicates emotional information, successfully engaging the viewers’ feelings. This heightened emotional engagement enhances the audience’s sense of involvement and overall viewing experience.

To measure emotional resonance, we employed the Self-Assessment Manikin (SAM) scale, focusing specifically on the dimensions of Arousal and Dominance. We excluded the Valence dimension as it may not be suitable for capturing emotional responses to all types of content, particularly those evoking complex or negative emotions. Participants rated their emotional states on a 9-point scale for each dimension. On the Arousal scale, a score of 1 indicated that viewers felt very calm, relaxed, or indifferent, while a score of 9 represented feeling highly excited, stimulated, or tense. For the Dominance dimension, a score of 1 suggested that viewers felt completely controlled by or submissive to the program’s emotional context, whereas a score of 9 indicated feeling entirely in control or dominant over the viewing experience. To ensure data reliability, we calculated the average score for each dimension across multiple assessments during the viewing session. The overall emotional resonance score was then derived by combining the mean scores of both dimensions with equal weighting, providing a comprehensive assessment of the viewers’ emotional engagement.

**Figure 5:**
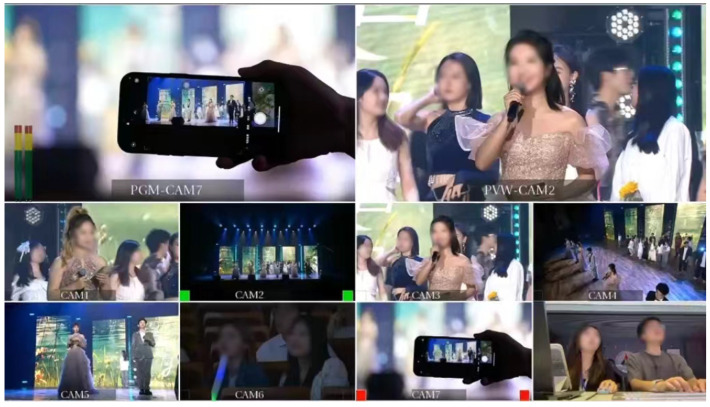
Broadcast control room display during the program performances. The figure shows the live monitoring of camera switching effectiveness across different shooting angles for the same stage performance.

During the broadcast test, we continuously monitored the effectiveness of camera switching, as shown in Figure 5, by recording the switching situations in the three types of programs. We provided timely feedback on the decisions made and obstacles encountered. Following the test, we reviewed and evaluated the overall performance of the EmotionCast system using subjective feedback from the audience and objective data analysis.

## 5. Results and Discussion

**Figure 6:**
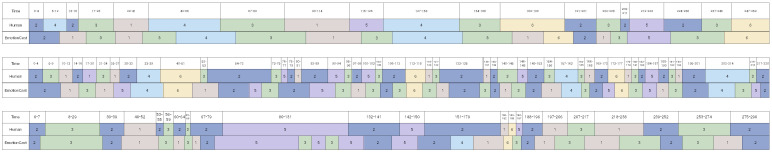
Visualization of camera switching timestamps for EmotionCast and human directors in a program. The moments of camera switching and the duration of each shot are shown. Different colors represent different camera positions.

We evaluated the performance of the system based on the test results, demonstrated the effectiveness of emotion-driven decision-making and consequently discussed the limitations encountered during implementation. The camera-switching timestamp chart visualizes the selection of different cameras by EmotionCast and human directors at various time intervals, using distinct colors to represent different camera positions. It records the moments of camera switching and the duration of each shot within a single program, as shown in Figure 6. This visualization aids in understanding the timing and consistency of the switches. Overall, EmotionCast’s switching decisions are closely aligned with those of the human directors. However, because on-site directors sometimes pursue personalized effects, they may choose shots with less visual information for variation and dynamically command real-time camera movements. Consequently, while ensuring the complete presentation of the program content, EmotionCast showed an approximately 15% difference from human selections.

By evaluating the performance of EmotionCast and human directors across three different types of programs—song, dance, and drama—we compared objective metrics, such as the number of switches, average switching interval, and standard deviation. The effectiveness of EmotionCast was demonstrated using all data presented in Table 1. In terms of switching frequency, EmotionCast performed better for slow-paced and narrative programs, maintaining longer durations in the same shot to convey the flow of emotions in the songs. For fast-paced action programs, the system tended to prefer a single type of shot and lacked balance among multiple camera angles. However, it maintained a similar number of switches as human directors, indicating its suitability for dynamic content. In terms of the average switching intervals, EmotionCast had shorter switching times. Further, compared with human directors, EmotionCast showed smaller differences between the longest and shortest intervals, and a smaller standard deviation of switching times, implying a more consistent switching pattern and providing a stable viewing experience.

**Figure 7:**
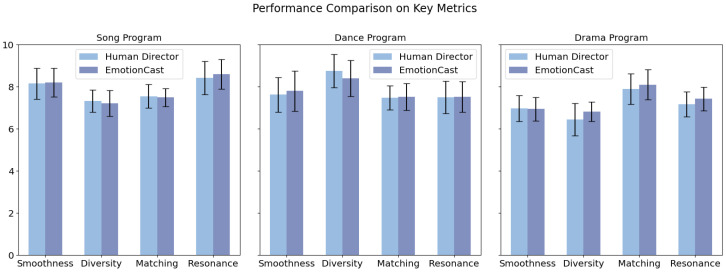
Visualization of subjective feedback from 20 participants on the three programs. The comparison between EmotionCast and human directors is based on four key parameters: smoothness, diversity, appropriateness, and emotional resonance. The Y-axis depicts mean ratings on a 10-point scale, where 10 represents maximum effectiveness. For uniformity in visualization, emotional resonance scores, initially measured on a 9-point scale, were proportionally adjusted to the 10-point scale used for other parameters. Error bars indicate the standard deviation of ratings, illustrating the range of participant responses. The ratings exhibit minimal differences between EmotionCast and human directors, indicating the system’s ability to deliver a comparable viewing experience.

For the subjective data collected through questionnaires, after verification and calculation of participant ratings, we derived final scores for four metrics: fluency, diversity, congruence, and emotional resonance. These results were visualized using Python, as shown in Figure 7. For all evaluated metrics, the differences in scores between EmotionCast’s automated decisions and those made by human directors were minimal, with variations consistently staying below 0.4 points. EmotionCast’s smoothness of switching score was close to that of human directors, indicating that it can provide a viewing experience comparable to that of human-directed broadcasts.

Regarding shot diversity, the largest gap between EmotionCast and the human directors occurred during the dance program. This was primarily owing to the large amplitude of movements in dance performances, where the system could not judge the camera motion, resulting in fewer rapid switches within short periods. Nevertheless, EmotionCast achieved high scores across all programs, indicating its ability to offer various engaging shot changes.

In terms of matching, EmotionCast outperformed human directors in both programs, demonstrating the system’s ability to effectively align with the program content needs. Feedback on emotional resonance exhibited excellent ratings of EmotionCast. Compared to human directors who focus on highlighting significant events, the emotion-driven directing style of EmotionCast enhanced the emotional expression of live content.

Overall, EmotionCast achieved a switching effect similar to that of human directors in both objective and subjective metrics, with superior performance in emotional presentation, adaptation to different content types, and the enhancement of emotional resonance. However, significant gaps remain between EmotionCast and human directors when addressing highly complex and subtle emotional scenes. The system requires improvements in recognition accuracy and response speed. In scenarios involving multiple interactions or rapidly alternating emotions, the system could not adjust the camera angles promptly, resulting in delays or misjudgments and potentially missing key emotional expressions.

Currently, AI-assisted performance evaluation is becoming a prominent research focus. The industry increasingly anticipates the development of more precise and objective evaluation tools. Our research aligns with this trend, offering new approaches to achieve this goal by integrating subjective scoring with objective indicators. For model training, the dataset used in EmotionCast is sourced from 10 gala recordings, totaling 25 h, from Communication University of China, encompassing a range of performances including singing, dancing, and spoken arts. During the training process, we ensured data validity by preprocessing all the data and leveraging expert evaluations to establish robust assessment standards. This approach was designed to guarantee that the training was comprehensive and effective, thereby enhancing the accuracy and reliability of the evaluation.

In conclusion, EmotionCast demonstrates the significant potential of emotion-driven directing technology for enhancing live broadcast quality and audience experience. Through continuous technological advancements and more in-depth scenario testing, this system is expected to be widely applied in the future to live broadcasts of various large-scale events, such as sports competitions and concerts, enhancing the audience’s sense of immersion and engagement. Additionally, the system can assist creative professionals and performers in refining their productions, particularly in enhancing emotional expression and audience resonance, bringing new transformations to the media industry.

## 6. Conclusions and Future Work

This study proposed EmotionCast, an innovative emotion-driven intelligent directing system designed to optimize directing decisions through real-time emotional analysis. The EmotionCast system design fully incorporated multimodal data fusion, emotion capture and recognition, and dynamic planning strategies. By integrating visual, audio, and textual data, EmotionCast utilizes advanced sensing technology to achieve emotion-driven camera switching. The test results showed that the EmotionCast system could effectively capture key emotional moments and promptly switch cameras, thereby enhancing the audiences’ emotional resonance. However, there is still room for improvement in the recognition of complex emotional states and optimization of switching frequency.

In future studies, we plan to focus on several areas. First, we will establish large-scale training datasets that cover more diverse scenarios and environmental data to improve the system’s generalization ability and verify its adaptability and robustness under different live broadcasting environments. Furthermore, addressing the technical challenges of future multimodal data fusion requires an in-depth exploration of more effective methods for integrating visual and auditory signals for emotion recognition. For example, combining attention mechanisms and transfer-learning techniques improves the accuracy and robustness of data fusion and emotion detection. With further optimization and enhancement, EmotionCast is expected to play a significant role in practical applications. Moreover, we plan to extend this technology to other types of media in the future. As it is further applied in sensing environments, EmotionCast is expected to play a significant role in various fields, driving innovation in media production and live broadcasting industries. In the future, we also plan to expand this technology to other types of media production.

## Figures and Tables

**Table 1:** Comparison of objective metrics between EmotionCast and human directors across three different programs. The number of switches, average switching intervals, and standard deviations are shown. The data highlight EmotionCast’s effectiveness and consistency in camera switching compared to human directors.

Evaluation Indexes	Song Program (259 s)		Dance Program (220 s)		Drama Program (290 s)	
	Human Director	EmotionCast	Human Director	EmotionCast	Human Director	EmotionCast
Switch Count	18	18	48	41	22	29
Average Interval	13.63 s	13.63 s	4.49 s	5.24 s	12.61 s	9.67 s
Standard Deviation	7.46	6.40	3.48	2.23	11.27	6.64
Longest Interval	27 s	25 s	19 s	10 s	52 s	36 s
Shortest Interval	3 s	4 s	2 s	2 s	2 s	3 s
Range	24 s	21 s	17 s	8 s	50 s	33 s

## Data Availability

Data are contained within the article.
